# Ion-Conducting Redox-Active Polymer Gels Based on Stable Nitroxide Radicals

**DOI:** 10.3390/polym11081322

**Published:** 2019-08-07

**Authors:** Fadoi Boujioui, Jean-François Gohy

**Affiliations:** Institute of condensed Matter and Nanosciences (IMCN), Catholic University of Louvain (UCLouvain), Place L. Pasteur 1, 1348 Louvain-la-Neuve, Belgium

**Keywords:** organic radical batteries, nitroxide radicals, ionic liquids, copper-catalyzed azide-alkyne cycloaddition

## Abstract

Redox-active polymer networks based on stable nitroxide radicals are a very promising class of materials to be used in the so-called organic radical batteries. In order to obtain fast-charging and high power electrodes, however, excellent ionic conductivity inside the electrode material is required to allow easy diffusion of ions and fast redox reactions. In this contribution, we investigated redox-active poly(2,2,6,6-tetramethylpiperidinyloxy-4-yl methacrylate) chains cross-linked through ionic liquid-like 1,2,3-triazolium groups. Different networks were prepared in which the amount of cross-linker and the counter-anion associated to the 1,2,3-triazolium group were varied. The ionic conductivities of the different polymer networks were first measured in the solid state by electrochemical impedance spectroscopy at different temperatures, and an increased ionic conductivity was measured when 1,2,3-triazolium groups were present in the network. The effects of the chemical nature of the counterions associated to the 1,2,3-triazolium groups and of the crosslinking density were then studied. The best ionic conductivities were obtained when bis (trifluoromethane)sulfonamide (TFSI) counter-anions were used, and when the crosslinking density of the TFSI-containing gel was higher. Finally, those ion-conducting gels were loaded with free LiTFSI and the transference number of lithium ions was accordingly measured. The good ionic conductivities and lithium ions transference numbers measured for the investigated redox-active gels make them ideal candidates for application as electrode materials for either organic radical batteries or pseudo-capacitors energy storage devices.

## 1. Introduction

Organic radical batteries (ORBs) are an emerging class of energy storage systems. The major advantage of ORBs lies in the fact that they are combining decent energy densities, albeit lower than for traditional Li-ion batteries, to high power abilities that could compete with supercapacitors [1]. Moreover, ORBs beneficiate from the well-known characteristic features of organic materials in terms of solubility, flexibility and compatibility with various preparation processes, including spray-drying, ink-jet printing, and so on [2]. ORBs based on stable radicals, e.g., diphenylpicrylhydrazyl [1], galvinoxyl [3], phenoxyl [4], and nitroxide [5], are particularly promising, because of their high stability during cycling. Among them, the most popular system has been introduced by Nishide and coworkers and consists of nitroxide radicals incorporated in a methacrylate polymer, namely poly(2,2,6,6-tetramethylpiperidinyloxy-4-yl methacrylate), abbreviated as PTMA [6]. PTMA-based electrodes are used as cathodes and are characterized by a reversible redox process of 3.6 V relative to Li/Li^+^, high rate performances with an ultra-fast electron transfer process of 10^−1^ cm/s, and a cycling stability exceeding 1000 cycles [7,8,9,10]. However, PTMA-based cathodes need to be formulated with large amounts of conducting carbons additives, since the electrical conductivity of PTMA is far too low. Moreover, the corresponding electrodes are characterized by a typical capacity fading upon cycling, due to continuous leaking of PTMA into the liquid electrolyte. In order to overcome the solubility problem of PTMA into the electrolyte, several strategies have been explored over the last years, including increasing the molar mass of PTMA [11], cross-linking PTMA chains into a network [12], and by physically or chemically grafting PTMA chains onto the conductive carbons [13,14,15]. Although those strategies have proven their usefulness, it is important to keep good solubility of the PTMA chains, in order to allow good accessibility for the lithium salts to the nitroxide radicals, and to exploit the full potential of the ultra-fast redox reactions of the nitroxide groups. Indeed, upon oxidation, the nitroxide radicals are converted into positively charged oxoammonium cations, and charge compensation is then afforded by the counter-anions associated to the lithium salts. Therefore, the ideal strategy should end up with a PTMA-based material that combines insolubility in battery electrolytes, while allowing good ionic conductivity in the bulk of the cathode. Interestingly, those prerequisites can be met by gel materials, the network being able to provide structural integrity while an adequate swelling in the electrolyte solution can support a fast flux of ions. Moreover, the use of gel-like electrodes characterized by minimized changes in swelling ratios during oxidation/reduction cycles of the nitroxide radicals is clearly advantageous, since this will minimize the mechanical stress experienced by the electrodes during battery operation. Such interesting characteristic features are not met by other types of electrode materials. This is the reason why we decided to focus our attention on PTMA gels. More precisely, we propose in this contribution to develop a simple synthetic strategy, allowing the formation of PTMA gels in which the solvent is indeed the liquid battery electrolyte. In order to increase the ionic conductivity inside the gel, and to avoid sharp changes in gel swelling ratios due to important ion fluxes during charging/discharging of the battery, we suggested the introduction of ionic triazolium crosslinking nodes mimicking ionic liquid moieties in the PTMA network. From a practical point of view, we set about to synthesize a crosslinker incorporating the 1,2,3-triazolium group. This crosslinker was then to be used to prepare a PTMA network that would be finally swelled with a liquid electrolyte to afford a PTMA ionic gel whose ionic conductivity would be measured by electrochemical impedance spectroscopy. The effect of the amount of crosslinker as well as the chemical nature of the counter-anions associated to the 1,2,3-triazolium cations would be investigated in detail. Finally, those gels were to be loaded with free lithium salt, and the transference number for lithium ions would be accordingly determined.

## 2. Materials and Methods

### 2.1. Materials

All reagents were used as received. 2,2,6,6-tetramethylpiperidin-4-yl methacrylate (TMPM, >98%) was purchased from TCI. Azobisisobutyronitrile (AIBN, >98%) and CuBr (>99%) were purchased from Acros. Ethylenediaminetetraacetic acid (EDTA, 99%), hydrogen peroxide solution (H_2_O_2_, 30% *w*/*w*), sodium tungstate dihydrate (>99%), *N*,*N*,*N*’,*N*’,*N*”-pentamethyldiethylenetriamine (PMDETA, 98%), ethylene carbonate (EC, >99%) diethyl carbonate (DEC, >99%), lithium hexafluorophosphate (LiPF_6_, battery grade, >99.99%), bis (trifluoromethane) sulfonimide lithium salt (LiTFSI_,_), methyl iodide (CH_3_I, >99%), and *N-*methyl bis [(trifluoromethyl) sulfonyl] imide (CH_3_TFSI, >90%), were purchased from Aldrich. The Porous polyethylene Celgard 2500 separator, diethylcarbonate (DEC), and ethylenecarbonate (EC) were purchased from MTI corporation.

### 2.2. Synthesis

#### 2.2.1. Synthesis of the 1,2,3-Triazole Cross-Linker

3-azidopropyl methacrylate (AzPMA) was synthesized as previously described [16]. The formation of AzPMA was confirmed by NMR.

^1^H-NMR (300 MHz, CDCl_3_), δ (ppm): 6.11 (m, 1H, C=CH**H**), 5.58 (m, 1H, C=C**H**H), 4.24 (t, 2H, C**H_2_**–O), 3.52 (t, 2H, C**H_2_**–N_3_), and 1.91–2.02 (m, 5H, overlapping C**H_3_**–C and C–C**H_2_**-C). ^13^C-NMR (300 MHz, CDCl_3_), δ (ppm): 167 (1C, C–**C**O_2_), 136 (1C, **C**=CH_2_), 125 (1C, H_2_**C**=C), 62.7 (1C, H_2_C–**C**-O), 46 (1C, N_3_–**C**H_2_), 29.5 (1C, H_2_C–**C**H_2_–CH_2_), and 17.9 (**C**H_3_–C).

Propargyl methacrylate (PMA) was synthesized with the following procedure [17]. In a 250 mL round-bottom flask, a solution of propargyl alcohol (11 mL, 185 mmol) and TEA (27 mL, 203 mmol) in CH_2_Cl_2_ (90 mL) was cooled in an ice bath. A solution of methacryloyl chloride (20 mL, 203 mmol) in CH_2_Cl_2_ (25 mL) was added dropwise over a period of 1 h, and the mixture was then stirred at room temperature overnight. The triethylammonium chloride salt was filtered and the mixture was extracted with an aqueous solution of hydrochloric acid (0.1 M), a saturated solution of NaHCO_3_, and water. The organic phase was dried over MgSO_4_. The crude product was purified by a silica gel column (hexane/ethyl acetate, 9/1) to afford a colorless oil.

^1^H-NMR (300 MHz, CDCl_3_) δ (ppm): 6.14 (m, 1H, C=C**H**H), 5.61 (m, 1H, C=CH**H**), 4.73 (s, 2H, OC**H_2_**), 2.46 (m, 1H, C≡C**H**), and 1.94 (m, 3H, C–C**H_3_**). ^13^C-NMR (300 MHz, CDCl_3_), δ (ppm): 167 (1C, C**C**O_2_), 137 (1C, **C**=CH_2_), 124 (1C, H_2_**C**=C), 77 (1C, HC≡**C**-CH_2_), 76 (1C, H**C**≡C), 52 (1C, ≡C-**C**H_2_-O-), and 17.9 (**C**H_3_–C).

Into a 50 mL round-bottom flask, PMA (500 mg, 4 mmol), AzPMA (680 mg, 4 mmol), and PMDETA (50 µL, 0.06 mmol) were dissolved in dichloromethane (25 mL, 95 wt%). The solution was bubbled with argon flux for 20 min, and CuBr (20 mg, 0.03 mmol) was added. The mixture was stirred at room temperature for 1 h before being heated at 37 °C for 24 h. The green solution was stirred under open air for 30 min and passed through neutral alumina column. The solvent was evaporated to give yellow viscous solid (0.98 g, 84%).

^1^H-NMR (300 MHz, DMSO-d_6_), δ (ppm): 8.21 (s, 1H, N–C**H**-C_quat_), 6.0 (m, 2H, 2*C=CH**H**), 5.6 (m, 2H, 2*C=C**H**H), 5.22 (s, 2H, C_quat_-C**H_2_**-O), 4.49 (t, 2H, N–C**H_2_**), 4.10 (t, 2H, O–C**H_2_**–CH_2_), 2.21 (m, 2H, H_2_C–C**H_2_**–CH_2_), and 1.87 (m, 6H, 2*C**H_3_**–C).

^13^C-NMR (300 MHz, DMSO-d_6_), δ (ppm): 166.4 (2C, 2*C–**C**O_2_), 141,8 (1C, **C_qua_**_t_–N), 135.6 (2C, 2*C=CH_2_), 126 (2C, C=**C**H_2_), 124.8 (1C, N–**C**H–C_quat_), 61.6 1C, H_2_C–**C**H_2_-O), 57.6 (1C, C_quat_–**C**H_2_–O), 46.7 (1C, N–**C**H_2_), 28.7 (1C, H_2_C–**C**H_2_–CH_2_), and 17.9 (1C, 2*H_3_**C**–C).

#### 2.2.2. Quaternization of 1,2,3-Triazole Cross-Linker

- Triazolium^+^/TFSI^−^ 1,2,3-triazole cross-linker (600 mg, 2.05 mmol) and *N-*methyl-bis[(trifluoromethyl) sulfonyl]imide (841 mg, 2.85 mmol) were mixed in acetonitrile (1 mL) and heated for 72 h at 41 °C. The crude compound was precipitated in diethyl ether and filtered on 0.22 µm poly(tetrafluoroethylene) (PTFE) filter. The obtained yellow solid was dried in vacuum at 40 °C (1.08 g, 90%).
- Triazolium^+^/I^−^ 1,2,3-triazole cross-linkers (260 mg, 0.887 mmol) and methyl iodide (1.26 mg, 8.87 mmol) were mixed in acetonitrile (1 mL) and heated for 72 h at 41 °C to ensure complete solubility. The crude compound was precipitated in diethyl ether and filtered on 0.22 µm PTFE filter. The yellow solid obtained was dried in vacuum at 40 °C (360 mg, 93%).

^1^H-NMR (300 MHz, DMSO-d_6_), δ (TMS, ppm): 9.05 (s, 1H, N–C**H**=C), 6.10 (d, 2H, C**H_2_**=C), 5.70 (d, 2H, C**H_2_**=C), 5.48 (s, 2H, C–C**H_2_**-O), 4.74 (t, 2H, CH_2_–C**H_2_**-N), 4.31 (s, 3H, N–C**H_3_**), 4.18 (t, 2H, O-C**H_2_**–CH_2_), 2.32 (quin, 2H, CH_2_–C**H_2_**–CH_2_), and 1.93 (d, 6H, 2*C**H_3_**–C).

^13^C-NMR (300 MHz, DMSO-d_6_), δ (TMS, ppm): 166.3 (d, 2*C=O), 138.6 (s, C=**C**-CH_2_), 135.6 (d, 2*C=CH_2_), 130.6 (s, **C**H_2_=C), 127.6 (s, **C**H_2_=C), 126.1 (s, N-**C**H_2_=C), 61.2 (s, C-**C**H_2_–O), 53.9 (s, O–**C**H_2_–CH_2_), 50.7 (s, CH_2_–**C**H_2_–N), 38.3 (s, N–**C**H_3_), 27.7 (s, CH_2_–**C**H_2_–CH_2_), and 17.9 (s, 2*CH_3_–C).

#### 2.2.3. Synthesis of PTMA Networks

In a Schlenk flask, TMPM (4 g, 17.7 mmol, 20 eq.) and triazole cross-linker (0.130 g; 0.444 mmol; 0.5 eq.) diluted in DMF (20 wt.%) were heated at 70 °C and bubbled with argon flux. Thereafter, a degassed solution of AIBN (0.030 g; 0.182 mmol; 0.2 eq. or 1% of reactants) in DMF was added in the Schlenk. The mixture was stirred at 70 °C overnight. The network was swollen in DCM to form a gel, was filtered on 0.5 µm PTFE filter and dried in vacuum at 40 °C. Once the PTMPM network was dried, it was oxidized into a PTMA network, following a previously described procedure [15]. More precisely, a round-bottomed flask equipped with a condenser was filled with PTMPM network (2.32 g, 10.3 mmol of amine functions, 1 eq.), Na_2_WO_4_.2H_2_O (0.849 mg, 2.6 mmol, 0.25 eq.), and EDTA (0.347 g, 1.5 mmol, 0.15 eq.) and methanol (120 mL). The solution was stirred at 60 °C for 5 min, and H_2_O_2_ (1.17 mL, 10.3 mmol, 10 eq.) was added dropwise over 60 min. The solution was stirred at 60 °C for 24 h. The polymer was extracted with CH_2_Cl_2_. The organic phase was dried with MgSO_4_. The solvent was removed under reduced pressure. The residue was precipitated in hexane, filtered and dried in vacuo at 30 °C overnight, affording an orange solid.

#### 2.2.4. Anion Exchange of Triazolium^+^/I^−^ PTMA Network into Triazolium^+^/PF_6_^−^ PTMA Network

Triazolium^+^/I^−^ based PTMA gel (0.50 g, 0.097 mmol) was swollen with acetonitrile (3mL) and the medium was bubbled with argon. Lithium hexafluorophosphate (0.026 mg, 0.174 mmol) was dissolved in acetonitrile (1 mL) in an argon glove box and added to the PTMA gel solution. Then, the medium was heated for 78 h at 40 °C. The product was added to 50 mL of methanol and filtered using a 0.22 µm PVDF filter. The gel was dried in vacuum at 40 °C.

#### 2.2.5. Sample Preparation for Electrochemical Impedance Spectroscopy (EIS) Measurements

**Figure 1:**
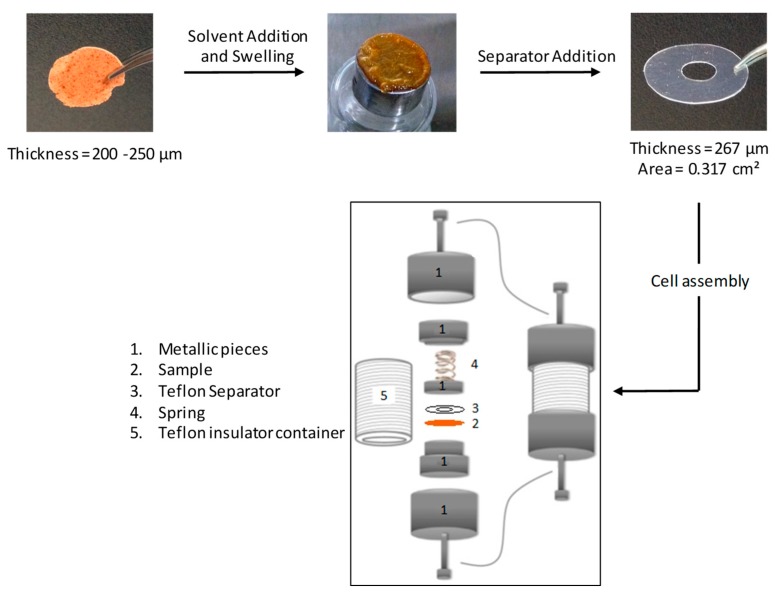
Sample preparation and Swagelok cell assembly for electrochemical impedance spectroscopy (EIS) measurements.

The PTMA network powders with different types of anions were pressed in pellets under 5 tons for a few minutes to obtain a thickness between 200 to 250 µm. The pellets were deposited on stainless steel electrodes and solvent (EC/DEC 1/1 vol/vol mixture) was added drop-by-drop until reaching a swollen state of gels. The thickness was kept constant with a 270 µm thick PTFE spacer between two stainless steel electrodes into a Swagelok cell (Figure 1). For the transference number measurements, the swelling of the networks, as well as the assembly of Swagelok cells were carried out in an argon glove box. All the cells rested 24 h before each measurement.

### 2.3. Instrumentation

#### 2.3.1. Nuclear Magnetic Resonance (NMR)

NMR spectra were acquired on a 300 MHz Bruker Avance II in DMSO-d_6_ or CDCl_3_.

#### 2.3.2. Electrochemical Impedance Spectroscopy (EIS)

EIS measurements were performed using the Parstat 4000 apparatus over a frequency range of 1 MHz to 10 mHz and with an amplitude AC excitation voltage of 5 mV. The temperature dependence measurements were carried out from 25 to 80 °C by gradually increasing the temperature by steps of 10 °C, with an equilibration time of 1 h between each step.

#### 2.3.3. Transference Number Measurements

They were carried out using Li|celgard|PTMA gel|celgard|Li symmetric cells assembled in an argon glove box. The DC polarization measurements were performed on an Arbin Instruments battery tester BT-2043 with a DC bias of 5 mV. The electrochemical impedance spectroscopy (EIS) measurements were performed on a Parstat 4000 with an AC bias of 5 mV.

## 3. Results and Discussion

### 3.1. Synthesis of the PTMA Gels Incorporating the 1,2,3 Triazolium Groups

**Figure 2:**
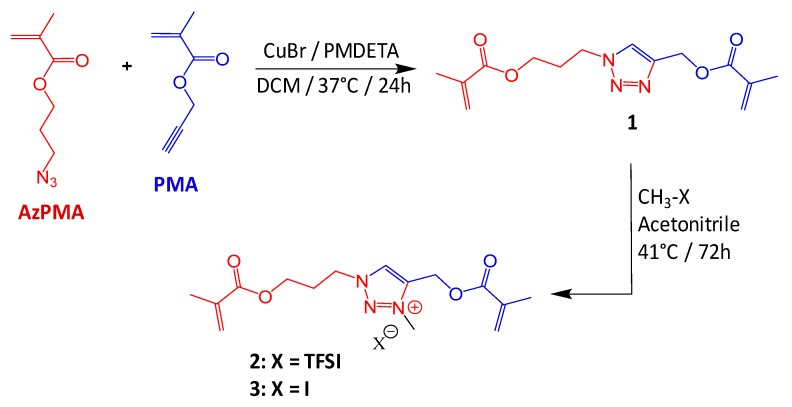
Synthesis of triazole (**1**), triazolium^+^/TFSI^−^ (**2**), and triazolium^+^/I^−^ crosslinker (**3**).

**Figure 3:**
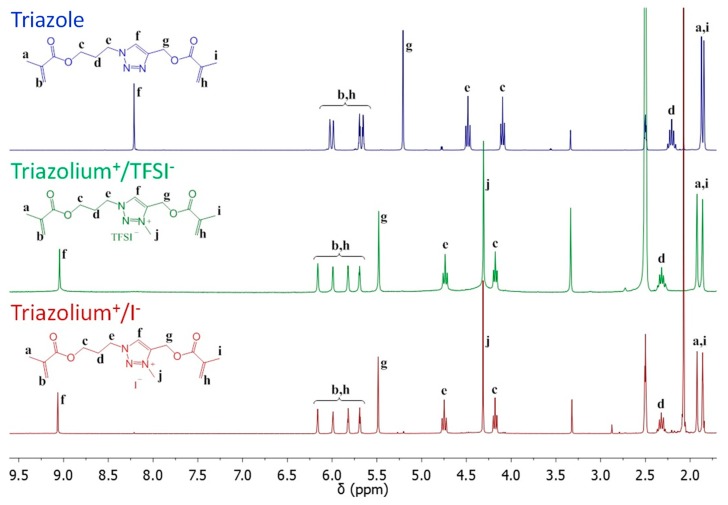
^1^H NMR spectrum in DMSO-d_6_ of triazole (on the top in blue), triazolium^+^/TFSI^−^ (on the middle in green) and Triazolium^+^/I^−^ (on the bottom in red) crosslinking nodes.

First, the 1,4-disubstituted 1,2,3-triazole crosslinking agent (**1** in Figure 2) was synthesized by the copper(I)-catalyzed alkyne-azide cycloaddition (CuAAC) reaction of 3-azidopropyl methacrylate (AzPMA) with propargyl methacrylate (PMA) catalyzed by Cu(I). According to NMR results (Triazole in Figure 3), the absence of characteristic peaks of azide and alkyne groups in the reaction product point towards a complete CuAAC reaction. Moreover, the observation of narrow peaks and the presence of vinyl peaks (in the crude samples after the removal of the copper) indicate that the use of dichloromethane at a low temperature has prevented the polymerization of methacrylate groups.

Then, the 1,2,3-triazole group of **1** was further alkylated using methyl bis [(trifluoromethyl) sulfonyl] imide and methyl iodide to provide ionic triazolium (**2**,**3**, respectively in the Figure 2). In order to allow a good solubilization of charged species, and therefore to promote the quaternization of the 1,2,3-triazole into 1,2,3-triazolium, a polar aprotic solvent, namely acetonitrile, was used. This reaction lead to a complete quaternization of the triazole group (Figure 3) as indicated by the shift toward the deshielded area of the characteristic triazole ring proton (f from 8.21 to 9.05 ppm) and by the appearance of a methyl group onto the triazole ring (j at 4.31 ppm).

**Figure 4:**
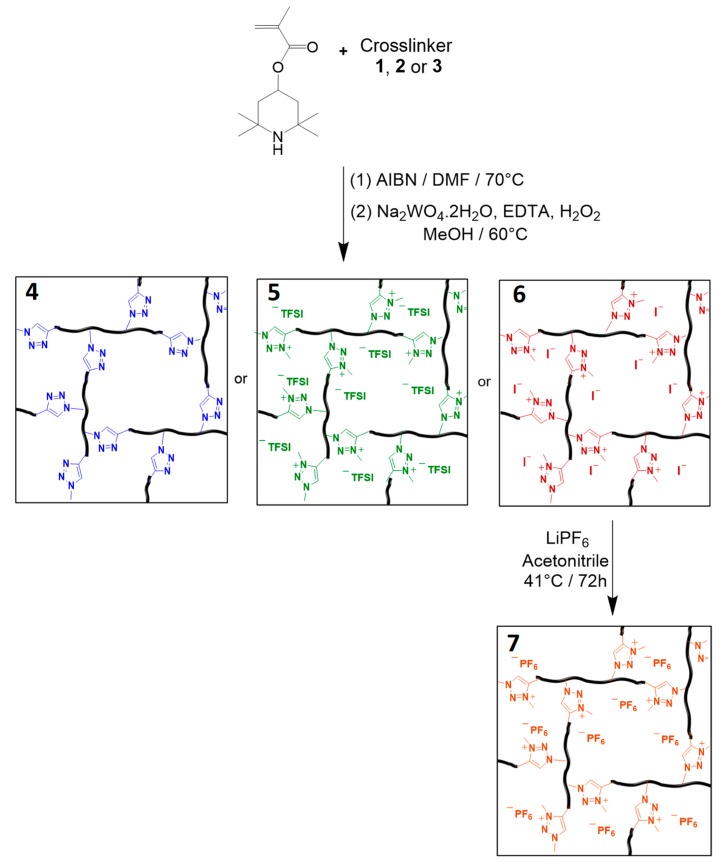
Synthesis of PTMA gels with different crosslinkers, (**4**) triazole gel, (**5**) triazolium^+^/TFSI^−^ gel, (**6**) Triazolium^+^/I^−^ gel and (**7**) triazolium^+^/PF6^−^ gel.

The synthesis of the PTMA gels was carried out via free radical polymerization way using 2,2,6,6-tetramethylpiperidin-4-yl methacrylate (TMPM) as the monomer, 1,4-disubstituted 1,2,3-triazole/triazolium as crosslinking agent (**1**–**3** of Figure 2) and AIBN as the initiator (Figure 4). After complete jellification and purification, the three PTMPM gels (triazole, triazolium^+^/TFSI^−^, and triazolium^+^/I^−^) were oxidized into PTMA gels with Na_2_WO_4_/H_2_O_2_ at 60 °C (**4**–**6** in Figure 4). This mild oxidation reaction condition is known to convert up to 99% of PTMPM secondary amines into nitroxide moieties without leading to decomposition of the polymer or network [18]. Moreover, the results shown below will also prove that this oxidation has no impact on the triazole and triazolium crosslinking nodes of PTMA networks. However, the last network named triazolium^+^/PF6^−^ (**7**) was obtained by anion exchange of Triazolium^+^/I^−^ network with LiPF_6_ salt dissociated in acetonitrile. This reaction was realized under a controlled atmosphere, after the oxidation of the PTMPM, to minimize or prevent the PF_6_^−^ anion degradation.

These four types of systems were synthesized according three different crosslinking degrees (A, B, and C) summarized in Table 1.

### 3.2. Influence of the Chemical Nature of the Crosslinking Nodes on the Ionic Conductivity

In order to investigate the influence of the chemical nature of the crosslinking nodes, the four systems (**4**–**7** in Figure 4) crosslinked at 2.4 mol % were investigated (crosslinking type B in Table 1). electrochemical impedance spectroscopy (EIS) has been used to evaluate the ionic conductivity of the different systems and has been practically performed at an AC voltage of 5 mV on the four networks sandwiched between two blocking stainless steel electrodes in a Swagelok cell (Figure 1). First, EIS measurements have been conducted on the dried PTMA networks with a 270 µm thickness, giving unfortunately, no signal. However, Boudouris et al. performed a similar analysis on a thin film of linear PTMA in the solid state, and they found a conductivity of ca. 1 × 10^−6^ S cm^−1^ [19]. Based on their work, we assume that the motion of PTMA chains is hindered by the presence of crosslinking nodes in our case. Then, the PTMA networks were studied in the gel state; that was achieved by swelling the networks with an ethylene carbonate (EC)/diethyl carbonate (DEC) solvent mixture (1/1 vol/vol). EC and DEC can be seen as plasticizers promoting the mobility of charge carriers by diffusing into the polymer network, thus increasing the free volume [20]. Moreover, they are good solvents for PTMA chains, as demonstrated by the solvent content reached at the equilibrium by the gels obtained by swelling the investigated PTMA networks in EC/DEC 1/1 vol/vol (Table 2). Indeed, a solvent content of c.a. 83 wt.% has been noted for the gel prepared from the neutral PTMA network containing triazole crosslinker, and a value as high as 95 wt.% for the gel containing charged crosslinking nodes. Although the presence of ionic species enables the PTMA gel to incorporate more solvent, the solvent content was kept constant (i.e., ca. 83 wt.% of solvent) for the EIS measurement of the four networks. No extra salt was added for these measurements since the first goal here was to determine the intrinsic conductivity of the PTMA gels.

**Figure 5:**
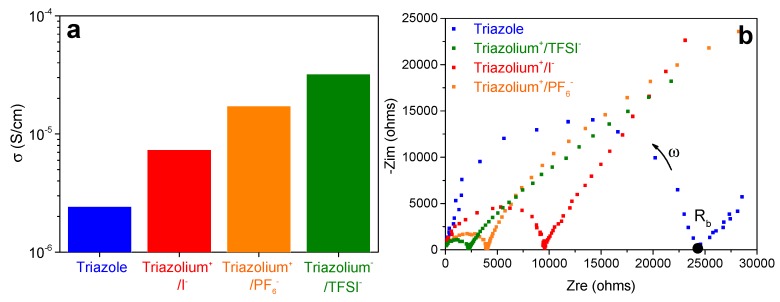
(**a**) Conductivity values and (**b**) Nyquist diagram of each gel comprising 2.4 mol % of crosslinker and swollen with EC/DEC (1/1). The measurements were carried out at ambient temperature, on cells composed of Stainless Steel|PTMA gel|Stainless Steel.

Figure 5a shows the conductivity (*σ*) of the investigated gels at ambient temperature. These conductivities were calculated using the following Equation (1):

(1) $$\sigma =L/(R_{b}\cdot A)$$

where *R_b_* is the resistance obtained from the Nyquist diagram in Figure 5b, *L* is the width, and *A* is the area of the sample in contact with the electrodes. The triazole gel (in blue) displays a conductivity of 2.4 × 10^−6^ S/cm. A similar value has been previously obtained for long chains of PTMA spin coated onto an ITO substrate [19]. For this system, the charge carriers were electrons belonging to nitroxide radical groups of PTMA. Thus, this electrical conductivity value is associated to the electron hopping mechanism along the PTMA chains facilitated by the liquid mixture of EC/DEC in which the PTMA chains are swollen. When the neutral triazole crosslinking nodes are replaced by charged triazolium^+^/X^−^ ones, the conductivity increases. The presence of counter-anions associated to the triazolium groups opens the possibility to observe ion mobility, and thus provides ionic conductivity, in addition to the electrical one generated by PTMA. The increase of conductivities follows the order: I^−^ (7.4 × 10^−6^ S/cm) < PF_6_^−^ (1.7 × 10^−5^ S/cm) < TFSI^−^ (3.2 × 10^−5^ S/cm). This is similar to the trend noted for swelling ratios and inversely related to the size of the anions (Table 2). Indeed, it has been reported that bulky anions generate an important charge delocalization enhancing the dissociation of triazolium^+^/X^−^ ions, and thus increasing the mobility of the counter-anions [22].

**Figure 6:**
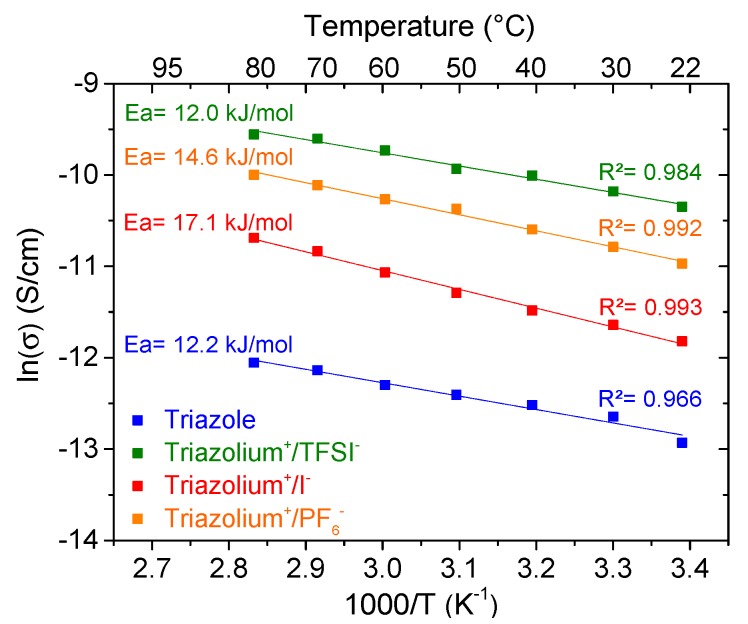
Temperature-dependent conductivity of each gel swollen with EC/DEC (1/1) and comprising 2.4 mol % of crosslinker. The cells are composed of stainless steel|PTMA gel|stainless steel.

The conductivities of the gels were then measured at variable temperature and plotted in Figure 6. For all investigated gels, the plots of ln (σ) versus 1000/T followed linear regression, indicating an Arrhenius behavior (Equation (2)).

(2) $$\sigma =A\cdot exp\left(-E_{a}/R\cdot T\right)$$

where *A* is a pre-exponential constant, *E_a_* is the activation energy for the conduction mechanism, *R* is the universal gas constant (8.314 J mol^−1^ K^−1^), and *T* is the temperature.

This Arrhenius behavior can be explained by the fact that in those experiments, the temperatures of measurement are always lower than the glass temperature of the PTMA which is estimated to be around 170 °C in bulk [19,23]. Moreover, the investigated gels can be seen as solid polymers interpenetrated by liquid, considering them as “semi-solid hybrid systems.” Thus, compared to linear chains, the segmental relaxation of the crosslinked chains in our systems is frozen, allowing the secondary relaxation (corresponding to the side chain group motion) to be the principal process.

Arrhenius fit of data gives an activation energy (*E_a_*) of 12.2 kJ/mol for the triazole (Figure 6), which can be attributed to the thermally activated electron hopping of TEMPO groups with the help of solvent. The *E_a_* of the triazolium+/TFSI− gel is identical to that of the neutral triazole gel. In contrast, the *E_a_* increases if the counter-ion is replaced by I^−^ (17.1 kJ/mol). This observation is in agreement with our previous results. In fact, among the three used counter-ions, the iodine anion is the smallest and is thus more strongly associated to the triazolium cation, influencing the energy barrier of the PTMA gel. In contrast, the TFSI^-^ anion is more mobile due to its weak association with the triazolium cation and has therefore no effect on the *E_a_* of the PTMA gel.

### 3.3. Influence of the Crosslinking Degree on the Ionic Conductivity

The effect of the crosslinking degree on the conductivity was also studied by varying the concentration of crosslinker in each investigated gel; i.e., 0.6 mol %, 2.4 mol %, and 9 mol %. For each set of cross-linked system, the solvent content was fixed based on the maximum swelling of their respective neutral triazole reference gel. Thus, all the gels crosslinked at 0.6 mol. %, 2.4 mol %, and 9 mol % were swollen with 93 wt.%, 83 wt.%, and 87 wt.% of solvent respectively.

**Figure 7:**
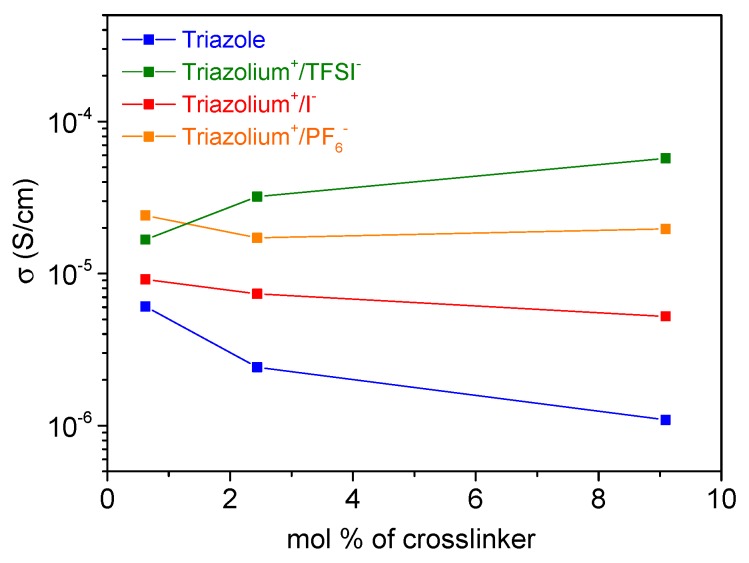
Conductivity values of each gel as a function of the molar percentage (mol %) of crosslinker. The cells are composed of stainless steel|PTMA gel swollen with EC:DEC (1:1)|stainless steel.

Figure 7 shows the conductivity of four PTMA networks swollen with EC/DEC solvent as a function of the crosslinker concentration (in mol %). Whatever the amount of crosslinker, the conductivity values follow the same trend as previously: *σ* triazole < *σ* triazolium+/I− < *σ* triazolium+/PF6− < *σ* triazolium+/TFSI−.

We can also observe that all gels reached a limit of conductivity around 1 × 10^−5^ S/cm when the crosslinker concentration approached zero. This conductivity value corresponds to the maximum value reached by a PTMA gel swollen with a maximum amount of EC/DEC solvent.

**Figure 8:**
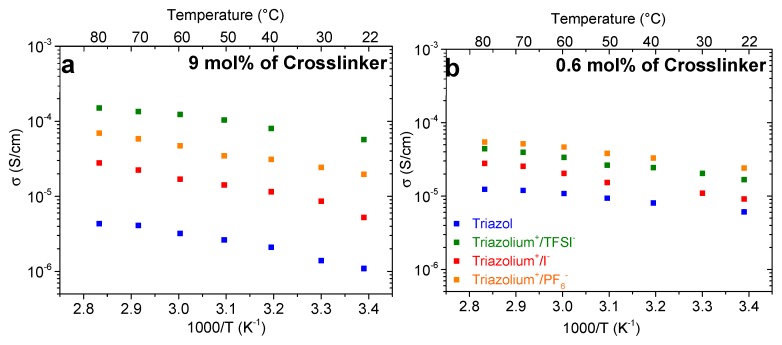
Temperature dependence of the ionic conductivity for PTMA gels comprised of (**a**) 9 mol %, and (**b**) 0.6 mol % of crosslinker.

The conductivity of the triazole gel decreased when increasing the amount of crosslinker. In fact, the gel comprising 0.6 mol % of triazole crosslinker displayed a conductivity value of 6.1 × 10^−6^ S/cm while 1 × 10^−6^ S/cm was measured for a concentration of 9 mol.%. A slightly cross-linked gel swells more and its side groups are more mobile, leading to a better conductivity. For the charged gels, such as the triazolium^+^/TFSI^−^ gel, an opposite effect is observed. Indeed, this gel comprising 9 mol % of triazolium^+^/TFSI^−^ crosslinker has a better conductivity than the less cross-linked counterparts reaching 5.7 × 10^−5^ S/cm. A large amount of triazolium^+^/TFSI^−^ crosslinker thus improves the conductivity of the PTMA gel, even though the latter is highly cross-linked, and swells less. A similar trend is observed with the increase of the temperature (Figure 8). The large TFSI^-^ anions are less strongly associated to the triazolium^+^ units than the other investigated anions and are thought to contribute importantly to the measured ionic conductivity. This could explain why the measured ionic conductivity values are the highest for the TFSI^−^ containing samples with higher cross-linking degree that contained a higher amount of mobile species.

**Figure 9:**
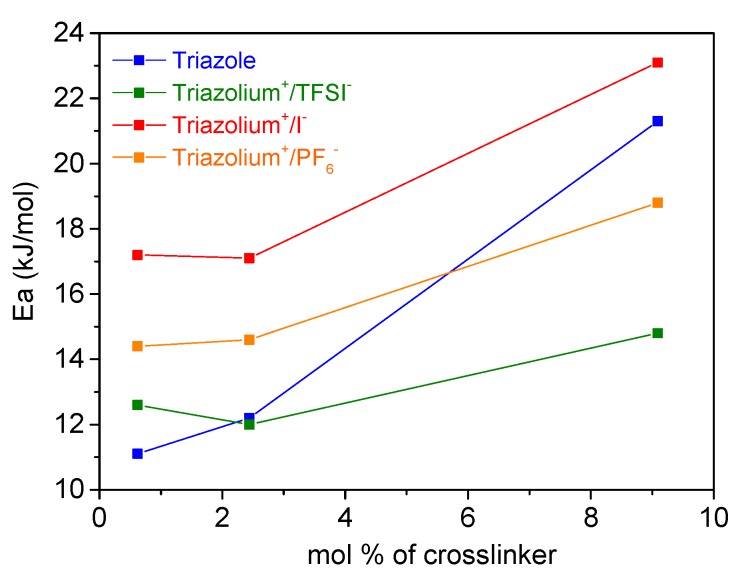
The plot of activation energy (*E_a_*) versus crosslinker concentration (in mol %).

The activation energies of all the investigated gels, obtained from the Arrhenius equation, increase with the crosslinker concentration (Figure 9). For the triazole gel, a 92% increase in the value of the *E_a_* is noted when the amount of crosslinker varies from 0.6 to 9 mol %. This influence on the energetic barrier can be explained by the poor solvent swelling and the poor mobility of the side groups of the PTMA chains. This increase is less pronounced with the presence of charged crosslinkers with 17% and 30% increase in the *E_a_* for triazolium^+^/TFSI^−^ and triazolium^+^/PF6^−^ gels, respectively. This result confirms the previously made hypothesis.

### 3.4. Loading of the Gels with Lithium Salts and Determination of the Lithium Ions Transference Number

The gels were also studied in the presence of added lithium salts. The four networks were, therefore, swollen with commercial electrolytes, such as LiTFSI (1M) in EC/DEC (1/1) and LiPF_6_ (1M) in EC/DEC (1/1). The solvent content of the gels was also kept constant here.

**Figure 10:**
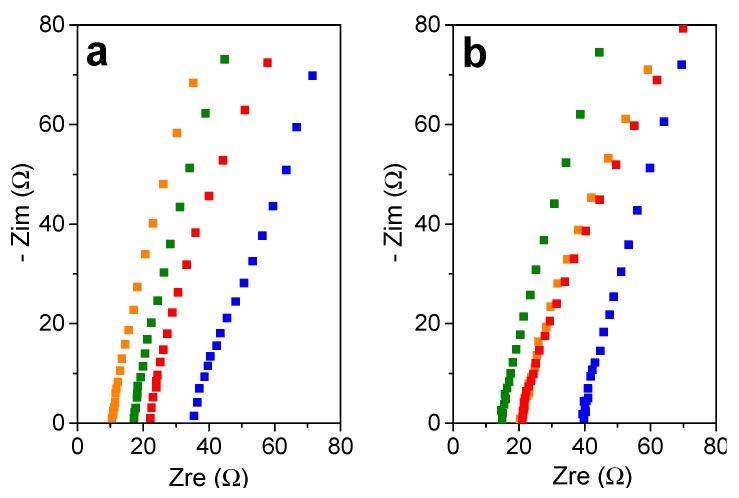
Nyquist diagram of triazole gel (in blue), triazolium^+^/TFSI^−^ gel (in green), Triazolium^+^/I^−^ gel (in red), and triazolium^+^/PF6^−^ (in orange). The gels were swollen with (**a**) LiPF_6_ (1M) in EC/DEC (1/1) and (**b**) LiTFSI (1M) in EC/DEC (1/1). The cells were composed of stainless steel|PTMA gel|stainless steel.

The swollen gels were again sandwiched between two stainless steel electrodes and EIS measurements were performed. Figure 10a,b shows Nyquist diagrams of swollen gels with LiPF_6_ and LiTFSI electrolytes, respectively. The obtained resistance values are much lower than those obtained previously in Figure 5b, as a result of the addition of lithium salts. In fact, the resistance was estimated at 35 and 40 ohms for triazole gels swollen with LiPF_6_ and LiTFSI in EC/DEC electrolytes respectively, while it reached 34.8 × 10^3^ ohms for the same network swollen with only EC/DEC (Figure 10b). Moreover, the charged gels showed better results again than the neutral triazole gel. Although, the resistance value of the triazolium+/PF6− swollen gel with LiPF_6_/EC/DEC electrolyte was slightly lower than these of swollen with LiTFSI EC/DEC electrolyte, the other charged gels kept the same trend as previously obtained, whatever the used solvent; i.e., *R_b_* (triazolium^+^/I^−^) > *R_b_* (triazolium^+^/TFSI^−^) > *R_b_* (triazole). We hypothesized that the presence of different counter-ions in the medium does not disturb the conductivity.

**Figure 11:**
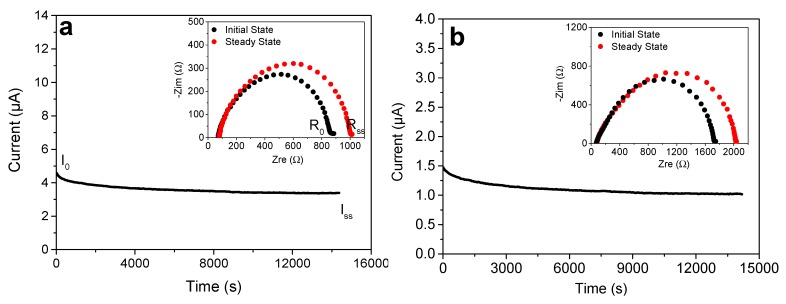
Time-dependence response of DC polarization for (**a**) Li|celgard|triazole gel with LiPF_6_-EC:DEC electrolyte|celgard|Li, and (**b**) Li|celgard|triazolium^+^/PF6^−^ gel with LiPF_6_-EC:DEC electrolyte|celgard|Li symmetric cell configuration at an applied DC bias of 5 mV and at 40 °C. The inset shows the impedance spectra of the initial and steady states of the cell with AC bias of 5 m.

The previously determined ionic mobilities include the contributions of both cations and anions movements. However, only the Li^+^ cations cross electrode interphases and participate in the intercalation process. The steady-state current approach developed by Bruce, Evans, and Vincent was then employed to study the mobility of lithium cations within the gels [24]. The swollen gels were sandwiched between two non-blocking lithium foils electrodes and a direct electric field (DC voltage of 5 mV in our case) was applied on the cell in order to polarize the lithium electrodes. At the positively polarized electrode, the Li was oxidized into *Li^+^*, which was reduced into *Li* at the negatively polarized electrode. Thus at *t* = 0, both cations and anions migrated under the DC voltage field, contributing to the ionic current. A steady-state equilibrium is reached when only Li^+^ cations, which are not blocked by the electrodes, contribute to the ionic current (Figure 11). The ratio of steady state current on initial current leads to the lithium transference number (*t_Li_^+^* = *I_ss_*/*I*_0_). However, Bruce, Evans, and Vincent noticed that the polarization of the cell leads to an increase of the resistance, corresponding to the increase of the passivation layer at the surface of metallic lithium. Thus, they reported that EIS measurements have to be conducted before and after the polarization in order to take into account the influence of the passivation layer, which is a consequence of the reaction electrolyte—lithium foil (insets of Figure 11). The combination of the DC voltage polarization and AC voltage impedance measurements (Figure 11) leads to the lithium ion transference numbers (*t_Li_^+^*) of both gels, by using Bruce–Evans–Vincent equation (Equation (3)):

(3) $$t_{Li+}=\;\frac{I_{ss}\left(\Delta V-I_{0}R_{0}\right)}{I_{0}\left(\Delta V-I_{ss}R_{ss}\right)}$$

where *I*_0_ and *I_ss_* are the initial and steady state currents obtained during the polarization; *R*_0_ and *R_ss_* are the resistances measured before (initial state) and after (steady-state) the polarization is applied; and Δ*V* is the DC potential bias.

The transference numbers were carried out on triazole (Figure 11a) and triazolium^+^/PF6^−^ (Figure 11b) swollen gels with LiPF_6_ (1M) in EC/DEC (1/1). Since PTMA is a weak intrinsic electrical conductor, celgard separators soaked with LiPF_6_ electrolyte were placed between lithium foils and the PTMA gels. The *t_Li_^+^* was estimated at 0.60 for the triazolium^+^/PF6^−^ gel and at 0.46 for the triazole gel. The mobility of lithium ions is thus facilitated in the charged PTMA network.

## 4. Conclusions

In this contribution, we have demonstrated that the conductivity and the transference numbers of lithium ions in PTMA-based gels are improved by the incorporation of charged groups mimicking ionic liquids as crosslinking nodes within the three-dimensional network. Moreover, we showed that the choice of the counter-anions, as well as the amount of ionic groups, corresponding to the crosslinker density, are very important for the proper functioning of the battery based on PTMA gel cathode. Without any added salts, the ionic conductivity in the PTMA-based gels is thought to be directly linked to the amount and to the mobility of ionic species in the system; i.e., triazolium cations and their associated anions. I^−^ and PF_6_^−^ anions strongly interact with triazolium cations, and the ionic conductivity increases when the cross-linking density decreases, because of the higher mobility of the chain segments in the less cross-linked gel. In sharp contrast, the PTMA gels containing triazolium^+^/TFSI^−^ crosslinking nodes exhibit higher ionic conductivities when the cross-linking density is higher; i.e., when the amount of triazolium^+^/TFSI^−^ units is higher. Since the bulky TFSI^−^ anions are less interactive with triazolium^+^ cations, the ionic mobility in those systems is directly linked to the amount of mobile TFSI^−^ anions, and the cross-linking density plays a minor role in that case. As expected, an increase in temperature results in an increased ionic conductivity for all the investigated PTMA-based gels. Finally, PTMA-gels were loaded with lithium salts in order to demonstrate the possibility to transport lithium ions. The transference number of lithium ions was increased in the ionic triazolium PTMA-gels compared to the neutral triazole PTMA-gel, demonstrating the interest of our strategy. Those gels will be investigated as cathode materials with fast charging/discharging abilities in a forthcoming paper.

## Figures and Tables

**Table 1:** Summary of three different crosslinking types.

Crosslinking Type	[Crosslinker]/[Monomer]	Mol % Crosslinking Agent
A	0.006	0.6
B	0.025	2.4
C	0.1	9

**Table 2:** Maximum EC/DEC solvent content (SC) and swelling ratio (SR) for the investigated gels as well as the volume of TFSI^−^, I^−^ and PF_6_^−^ anions.

Gel Sample	SC ^1^ (wt.%)	Anion Volume ^2^ (Å^3^)
4	83	-
5	86	34.31
6	93	72.61
7	95	147.65

^1^$\mathrm{SC}\;\left(\mathrm{wt}.\%\right)=\left[\left(W_{\mathrm{swollen}}-W_{\mathrm{dry}}\right)/W_{\mathrm{swollen}}\right]*100,$^2^ From reference [21].
